# Seroepidemiological survey to investigate *Rickettsia rickettsii* and *Rickettsia parkeri* in municipalities of the southeast Brazil

**DOI:** 10.1590/S1678-9946202668001

**Published:** 2026-01-30

**Authors:** Mariani Borges Franco, Gustavo Cardoso Fonseca, Ana Carolina Prado Sousa, Cristina Rostkwoska, Ana Cláudia Arantes Marquez Pajuaba, José Roberto Mineo, Matias Pablo Juan Szabó, Stefan Vilges de Oliveira

**Affiliations:** 1 Universidade Federal de Uberlândia Faculdade de Medicina Programa de Pós-Graduação em Ciências da Saúde Uberlândia Minas Gerais Brazil Universidade Federal de Uberlândia, Faculdade de Medicina, Programa de Pós-Graduação em Ciências da Saúde, Uberlândia, Minas Gerais, Brazil; 2 Universidade Federal de Uberlândia Faculdade de Medicina Veterinária Uberlândia Minas Gerais Brazil Universidade Federal de Uberlândia, Faculdade de Medicina Veterinária, Uberlândia, Minas Gerais, Brazil; 3 Universidade Federal de Uberlândia Faculdade de Medicina Veterinária Laboratório de Ixodologia Uberlândia Minas Gerais Brazil Universidade Federal de Uberlândia, Faculdade de Medicina Veterinária, Laboratório de Ixodologia, Uberlândia, Minas Gerais, Brazil; 4 Universidade Federal de Uberlândia Instituto de Ciências Biomédicas Laboratório de Imunoparasitologia Uberlândia Minas Gerais Brazil Universidade Federal de Uberlândia, Instituto de Ciências Biomédicas, Laboratório de Imunoparasitologia, Uberlândia, Minas Gerais, Brazil; 5 Universidade Federal de Uberlândia Faculdade de Medicina Departamento de Saúde Coletiva Uberlândia Minas Brazil Universidade Federal de Uberlândia, Faculdade de Medicina, Departamento de Saúde Coletiva, Uberlândia, Minas, Brazil

**Keywords:** Spotted fever, Rickettsia, Tick, Zoonosis

## Abstract

Spotted fever is a tick-borne rickettsiosis caused by several *Rickettsia* species—including *R. rickettsii, R. parkeri*, and others—with varying degrees of pathogenicity. Its nonspecific symptoms often lead to misdiagnosis such as dengue. This study investigated anti-*R. rickettsii* and *R. parkeri* antibodies in 152 patients with acute febrile illness who tested negative for dengue. Serological analysis using immunofluorescence assay found 29 reactive samples (19%) at a 1:64 dilution. Among them, 20.6% were male and 58.6% female, with an average age of 42.6 years. The average sample collection time totaled 14.6 days. Reactive samples included 13.1% for *R. rickettsii* and 5.9% for *R. parkeri*. These results suggest possible rickettsial infections in patients initially suspected of dengue.

## INTRODUCTION

Tick-borne diseases are a global public health concern. Their etiologic agents*, Rickettsia* spp., are obligate intracellular gram-negative bacteria that infect endothelial cells, leading to vasculitis and systemic inflammatory responses^1^. *Rickettsia rickettsii* and *Rickettsia parkeri* cause spotted fever, with confirmed cases in Brazil. Its symptoms are often confused with dengue after an incubation period of 2 to 14 days^2,3^.

Spotted fever due to *R. rickettsii* may present high fever, rash, and multiple organ dysfunction, carrying high lethality if untreated^12^. In southeast Brazil and northern Parana State, *Rickettsia rickettsii* is transmitted by *Amblyomma sculptum* and *Amblyomma aureolatum*, particularly in the metropolitan area of Sao Paulo State. On the other hand, *Rickettsia parkeri*, especially the Atlantic Rainforest strain, is associated with *Amblyomma ovale* in the Atlantic Forest and is linked to milder forms of the disease in Brazil^3,4^.

In the case of *Amblyomma sculptum*, human infections are more commonly associated with the immature stages of the species (larvae and nymphs), whereas adult *Amblyomma aureolatum* and *Amblyomma ovale* are primarily responsible for transmission^5^.

From 2007 to 2021, Brazil recorded 36,497 spotted fever notifications, averaging 170 confirmed cases per year^6^. Minas Gerais State ranks third in confirmed cases and second in fatalities, with a 32.8% lethality rate from 2007 to 2019, mostly affecting males aged 30–59 years with contact history involving ticks, capybaras, or domestic animals^7^.

Diagnosis relies on indirect immunofluorescence assay (IFA), immunohistochemistry, or polymerase chain reaction but due to the rapid progression of the disease, treatment should begin based on clinical-epidemiological suspicion before lab confirmation^8,9^.

Due to its nonspecific clinical presentation, especially in mild or oligosymptomatic cases, spotted fever is often misdiagnosed as more prevalent illnesses such as dengue^10^. Therefore, this study investigates the seroprevalence of antibodies to *R. rickettsii* and *R. parkeri* in the Triangulo Mineiro region of Minas Gerais State, targeting patients with acute febrile illness who tested negative for dengue.

## MATERIAL AND METHODS

Epidemiological data from 4,124 patients who were referred to the Laboratory of Immunoparasitology Dr. Mario Endsfeldz Camargo at the Federal University of Uberlandia for dengue screening in 2019 were included in this study. Via convenience sampling, 1,856 aliquots of blood serum with negative results for dengue were selected, of which 152 were subjected to serological analysis for *Rickettsia rickettsii* and *Rickettsia parkeri*. No active recruitment occurred; only anonymized secondary data were used.

Selection criteria prioritized samples from rural/peri-urban areas or outdoor workers (52) and those collected ≥14 days after symptom onset (100) to increase likelihood of *Rickettsia* exposure and IgG detection. Samples with missing epidemiological data or unsuitable storage conditions (hemolysis, lipemia, or low volume) were excluded. All selected samples were dengue-negative by IgM ELISA (PanBio™, Abbott).

Serum was tested by in-house IFA using Brazilian strains of *R. rickettsii* and *R. parkeri* that were provided by Prof. Marcelo B. Labruna from the Faculty of Veterinary Medicine and Zootechny of the University of Sao Paulo. The analyses were conducted at the Ixodology Laboratory of the Faculty of Veterinary Medicine at the Federal University of Uberlandia.

Sera were screened at 1:64 dilution using FITC-labeled anti-human IgG (1:600, Sigma), with positive and negative controls. Reactive samples were titrated in twofold dilutions and analyzed by epifluorescence microscopy at 40×.

### Ethics

This study was approved by the UFU Research Ethics Committee (process Nº 5.392.382) and complied with National Research Ethics Commission guidelines (Resolutions Nº 466, of December 12, 2012, and Nº 510, of April 7, 2016, of the National Health Council).

## RESULTS

Of the 152 analyzed samples, 29 (19%) were reactive at 1:64 dilution for *R. rickettsii* or *R. parkeri*. No reactivity occurred at higher titers. Most were female (58.6%), with an average age of 42.6 years and collection time of 14.6 days. Of the reactive cases, 41.3% were White, 27.6% Brown, and 24.1% Black individuals, mainly from Uberlandia (31%) and Araguari (20.6%). In total, 96.5% reside in urban areas.

Of the 29 reactive patients, one was hospitalized with severe symptoms (persistent vomiting, abdominal pain, fluid accumulation, and increase in hematocrit). This patient tested positive for *R. rickettsii* 18 days after symptom onset. Reported symptoms included fever, myalgia, rash, and vomiting, which are also common among other reactive cases (62% had fever/myalgia; 31%, vomiting; and 13.7%, rashes). Moreover, four other reactive cases involved rural or agricultural workers. These individuals engaged themselves in occupations related to potential exposure to *Rickettsia* sp.

Samples reactive at a dilution of 1:64, specifically for *Rickettsia rickettsii* and *Rickettsia parkeri*, represented 13.1% and 5.9% of all samples, respectively. Moreover, 20 samples were reactive to *R. rickettsii*, and nine to *R. parkeri*. Reactivity to *R. rickettsii* was higher among female patients. Of the 20 patients, 40% were aged from 18 to 40 years and 35% from 41 to 60 years. A total of 95% lived in urban areas. Table 1 shows their epidemiological profiles.

**Table 1:** Sociodemographic characteristics of patients with clinical suspicion of dengue, whose samples were reactive in serological tests for the diagnosis of *Rickettsia rickettsii* and *Rickettsia parkeri*, Triangulo Mineiro, Minas Gerais State, Brazil, 2019

		Reactive for *Rickettsia*sp.(1:64)	Reactive for *R. parkeri*(1:64)	Reactive for *R. rickettsia*(1:64)
**Number of reactive samples/Number of analyzed samples**		29/152 (19.8%)	09/152 (5.9%)	20/152 (13.1%)
**Sex**				
	Female	17	07	14
	Male	06	02	16
**Age (years)**				
	0–17	02	01	02
	18–40	09	02	08
	41–60	08	05	07
	60 and over	04	01	03
**Average age (years)**		42.6	45.8	42.1
**Average collection time (days)**		14.6	15	14
**Municipality of origin**				
	Uberlandia	09	03	08
	Araguari	06	02	06
	Monte Carmelo	02	02	01
	Grupiara	01	01	00
	Monte Alegre	02	00	02
	Patrocinio	01	00	01
	Nova Ponte	01	00	01
	Arapora	01	01	00
**Skin color**				
	White	12	06	09
	Mixed-race	08	03	08
	Black	07	00	01
	Ignored/Not informed	02	00	02
**Area**				
	Peri-urban/Rural	01	00	01
	Urban	22	09	19
**Hospitalizations**		01	00	01
**Education**				
	Illiterate	00	00	00
	Elementary school I	08	01	03
	Elementary school II	04	05	07
	High school	05	03	04
	Higher education	01	00	01
	Ignored/Not informed	05	00	05

## DISCUSSION

Spotted fever is endemic in Brazil and has expanded to rural, urban, and peri-urban areas, increasing exposure risk. Monitoring epidemiologiarel trends and identifying high-incidence regions is essential^1^. Early diagnosis is critical but challenging due to symptoms overlapping with other febrile illnesses and limited access to rapid testing^11^.

We found that 19% (29/152) of samples were reactive at a 1:64 dilution using IFA (the gold standard for the serological diagnosis of rickettsioses). Ideally, confirmation requires a fourfold or greater rise in IgG titters between paired serum samples collected at least 14 days apart^12^. However, due to the use of convenience sampling, only single samples were available, preventing definitive confirmation of spotted fever cases. Reactivity only at 1:64 dilution may suggest cross-reactivity with other *Rickettsia* species not primarily investigated. While *R. rickettsii* and *R. parkeri* are the main human pathogens in Brazil, other species, such as *R. rhipicephali, R. amblyommatis*, and *R. bellii* (the latter widely distributed and of uncertain pathogenicity), have also been reported^13^.

Previous serological surveys have highlighted cross-reactivity challenges in diagnosing spotted fever. Novo Cruzeiro, Minas Gerais State, showed a 10.1% seroprevalence for spotted fever agents, with 18% of sera showing IFA titers ≥1:64 for *R. rickettsii*, despite no symptoms in the preceding three years. The findings suggest a cross-reactivity that may have been caused by other *Rickettsia* species^14^. Another study in Piau, Minas Gerais, by Costa *et al*.^15^, reported frequent cross-reactivity among *R. rickettsii, R. typhi, Coxiella burnetii, Bartonella* spp., and *Ehrlichia chaffeensis*, likely due to shared antigenic determinants. The authors stressed the importance of simultaneously testing multiple rickettsial antigens to improve diagnostic accuracy. they noted no strong correlation between seropositivity and rural occupations. In contrast, our study found that four out of 29 reactive samples (13.7%) had come from individuals with outdoor professions, indicating a potential occupational risk.

Although most *Rickettsia* vectors, such as *Amblyomma sculptum, A. aureolatum*, and *A. ovale*, are typically associated with rural or peri-urban environments, most reactive cases in our study (96.5%) occurred in individuals residing in urban areas. Other Brazilian studies have found similar findings, suggesting that urban residents may still be exposed in visits to rural or natural areas for work or leisure or via peri-urban ecotones with vector-host interactions. Additionally, finding *Rickettsia* in urban cases may reflect increased mobility, underreporting of exposure history, or environmental changes that bring humans, reservoirs (e.g., capybaras, dogs), and vectors into closer contact. Note that *Rhipicephalus sanguineus* sensu lato, a species adapted to urban settings, has rarely been implicated as a vector of pathogenic *Rickettsia* species in Brazil^3^. These findings show the importance of considering spotted fever in patients from urban and non-endemic regions. *Rickettsia* sp. infections have been reported in dogs from non-endemic areas in southeastern Brazil, confirmed by serological and molecular analyses^16^. Given their outdoor habits and close human interaction, dogs serve as sentinels for SF transmission^3^.

The transmission of *Rickettsia* sp. to humans primarily depends on ticks, with *Amblyomma sculptum* and *A. aureolatum* configure as the main vectors in Brazil, and *Rhipicephalus sanguineus* considered a possible vector under specific conditions. However, current knowledge about tick vectors and their hosts, such as the capybara (*H. hydrochaeris)* remains limited, hindering a full understanding of the epidemiology of the disease. Accurate identification and mapping of tick species by region are essential to explain the transmission dynamics of rickettsioses. Moreover, the continuous discovery of new *Rickettsia* species and potential human infections highlights the complexity of their enzootic and epidemic cycles and the diversity of tick vectors. Rapid environmental changes driven by human activity further complicate these dynamics, reshaping host-vector interactions and potentially leading to new scenarios for tick-borne diseases^17^.

Human-driven environmental changes have increased interactions between humans and pathogens. In response, the One Health approach emphasizes collaboration between physicians, ecologists, and veterinarians, recognizing the interconnected health of humans, animals, and the environment. Sentinel animals such as horses and dogs have been used in spotted fever surveillance in endemic areas. Moreover, clinical suspicion should arise in any patient presenting symptoms such as fever, myalgia, headache, and rashes, especially in regions with high seroprevalence among sentinel species^18^.

Spotted fever presents symptoms such as fever, severe headache, nausea, vomiting, diarrhea, abdominal pain, muscle pain, limb paralysis, and gangrene, with red, non-itchy spots often appearing on wrists and ankles as the disease progresses. These manifestations resemble those of dengue, which include high fever, body aches, headache, rash, and warning signs such as abdominal pain, vomiting, and increased hematocrit^10^. In our study, one hospitalized patient showed such warning signs and was initially tested for dengue (non-reactive). However, serological analysis showed reactivity to *R. parkeri* (1:64), with reported symptoms of fever, myalgia, rash, and vomiting. In this study, of the 29 reactive samples, the most frequently reported symptoms included fever (62.07%), myalgia (62.07%), headache (58.62%), and back pain (44.83%), followed by gastrointestinal symptoms such as nausea (34.48%) and vomiting (31.03%). Although these signs are non-specific and overlap with other endemic febrile illnesses such as dengue, they are consistent with the clinical pattern of spotted fever in Brazil. According to Oliveira *et al*.^19^, fever, headache, myalgia, and prostration were the most prevalent symptoms among confirmed cases, reinforcing the relevance of these findings and the need for increased clinical suspicion in endemic or at-risk areas^19^. The results partially diverge from the epidemiological data in the literature, which indicates that rickettsiosis occurs more prevalently in adult men. However, of the 152 samples in this study, 85 (55.9%) came from women and 67 (44.1%) from men. Considering the greater representation of women in the sample, this may have influenced the higher proportion of reactivity in this group^19^.

Our findings showed rickettsial reactivity in patients initially suspected of having dengue, an endemic arbovirus in the region. This overlap may delay rickettsiosis diagnosis, highlighting the importance of evaluating patient history related to tick exposure. Given the high lethality of spotted fever, timely diagnosis is crucial. This study underscores the need to consider spotted fever in acute febrile cases, even at low antibody titers. The lack of paired samples and detailed patient exposure data limits deeper analysis, reinforcing the need for further epidemiological research in the region.

## CONCLUSION

The findings showed rickettsial reactivity in suspected dengue patients, highlighting the need of considering spotted fever diagnosis even in non-endemic areas. Interdisciplinary collaboration can enhance epidemiological research and control strategies. Educational measures, including training healthcare providers, public awareness campaigns, and tick control, should be promoted.

## Data Availability

The complete anonymized dataset supporting the findings of this study is included in this article.
